# Percutaneous transforaminal endoscopic discectomy for the treatment of lateral recess stenosis secondary occurred the discal fungus infection

**DOI:** 10.1186/s12891-020-03211-7

**Published:** 2020-03-18

**Authors:** Yunpeng Fan, Tao Xie, Yao Pang, Liulong Zhu, Shaobo Zhou

**Affiliations:** 1 The Affiliated Hangzhou Hospital of Nanjing Medical University, Hangzhou, 310006 China; 2 The affiliated Hangzhou First People’s Hospital, Zhejiang University School of Medicine Hangzhou, 261# huansha road, Shangcheng District, Hangzhou City, 310006 Zhejiang Province China

**Keywords:** Lumbar spine, Lateral recess stenosis, Intervertebral infection, Fungus, Percutaneous transforaminal endoscopic discectomy

## Abstract

**Background:**

This is a case of lateral recess stenosis secondary occurred the discal fungus infection treated with percutaneous transforaminal endoscopic discectomy (PTED). There has been no relevant reports before.

**Case presentation:**

A 49-year-old patient who had taken itraconazole for 13 months for lateral recess stenosis secondary occurred the discal fungus infection complained of gradually worsening radiating pain and numbness in the back and inguinal and inner thigh region of right side. In order to relieve the radiating neuralgia and reduce the damage to spinal stability, the minimally invasive PTED was performed.The patient’s prognosis was assessed using Oswestry Disability Index (ODI) and Visual Analogue Scale (VAS).

During the follow-up, the patient’s ODI and VAS scores were decreased significantly. The radiating pain in the inguinal and inner thigh region of right side were significantly alleviated and the discomfort caused by lower back instability was improved by plaster vest.

**Discussion and conclusion:**

PTED not only avoids further damage to the stability of the lumbar spine, but also effectively relieves the symptoms of leg neuroradialgia caused by lateral recess stenosis secondary occurred the discal fungus infection.

## Background

Lateral recess stenosis is the common disease in elder people [1]. However, intervertebral disc fungal infection is very rare because only people with immune deficiency will develop this infection [2, 3]. Early diagnosis and treatment of this infection is difficult because the symptoms are atypical and sometimes covered up by other diseases [4, 5]. Nowadays, open surgery is widely accepted for the treatment of this [6] because it can expose the segmental visual field of the focus. However, the tissues undergo open surgery are more prone to damage and the subsequent infection, whereas minimally invasive surgery can decrease damage and better avoid post-operative infection [7, 8]. Here we report a case of lateral recess stenosis secondary occurred the discal fungus infection treated with minimally invasive PTED in our hospital and evaluate the efficacy and feasibility of this technique.

## Case presentation

**Fig. 1:**
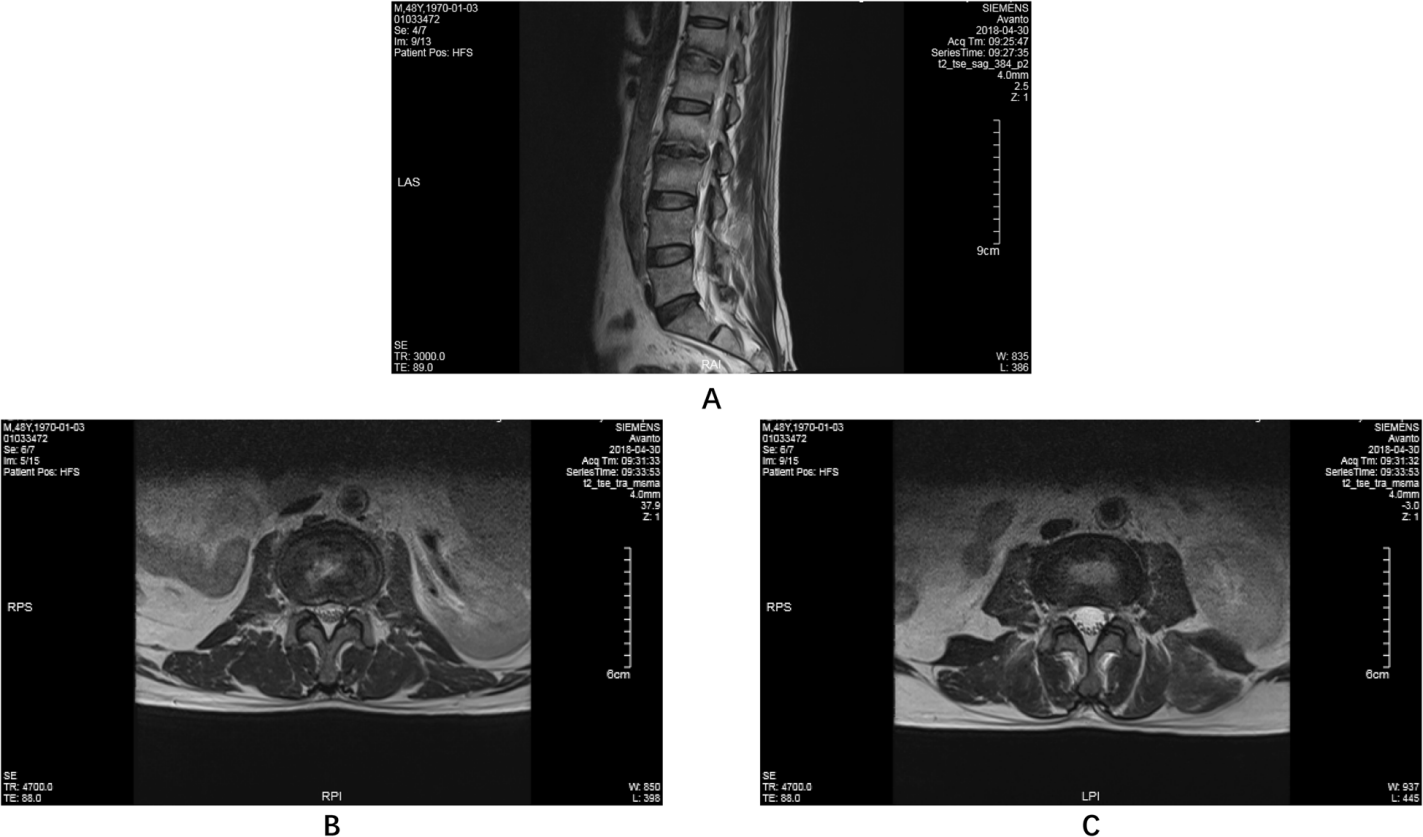
Lumbar MRI (2018-04-30) **a** sagittal plane. **b** transverse section of T12/L1 **c** transverse section of L2/L3

**Fig. 2:**
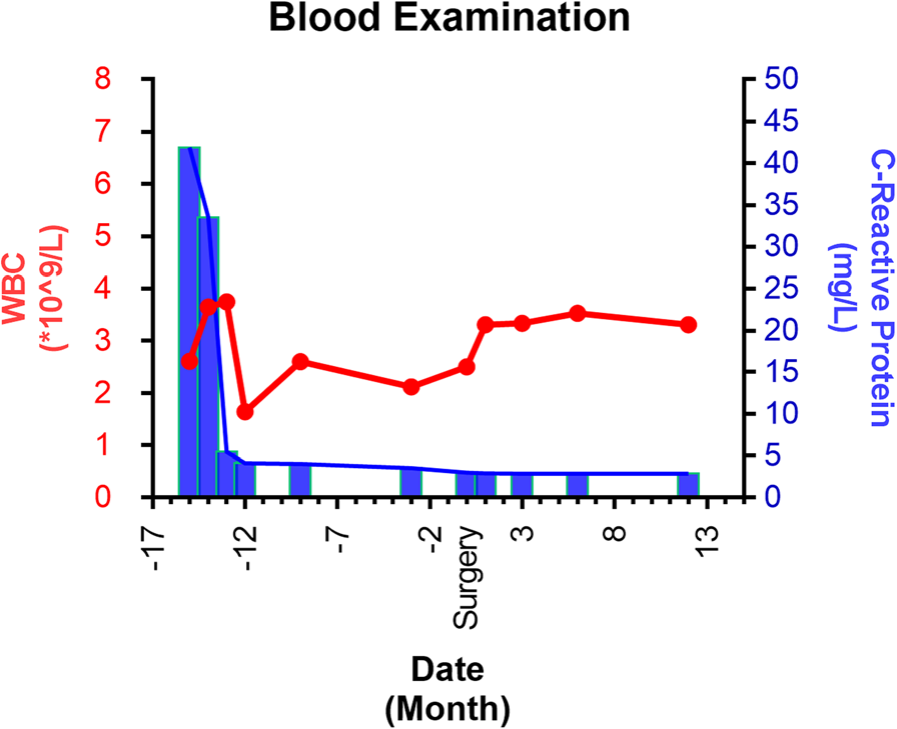
Blood examination result of WBC and CRP

**Fig. 3:**
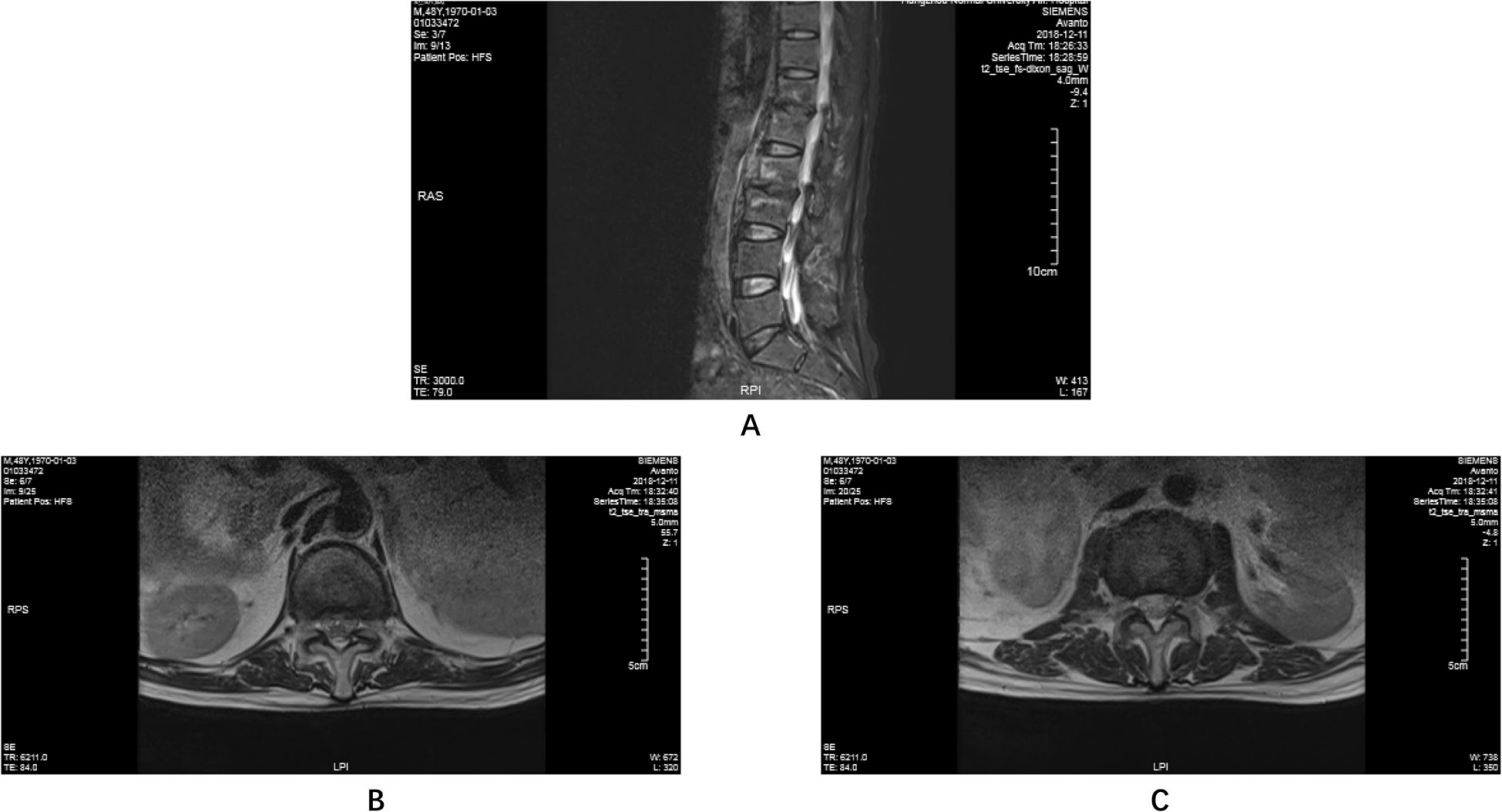
Lumbar MRI (2018-12-11) **a** sagittal plane. **b** transverse section of T12/L1. **c** transverse section of L2/L3

**Fig. 4:**
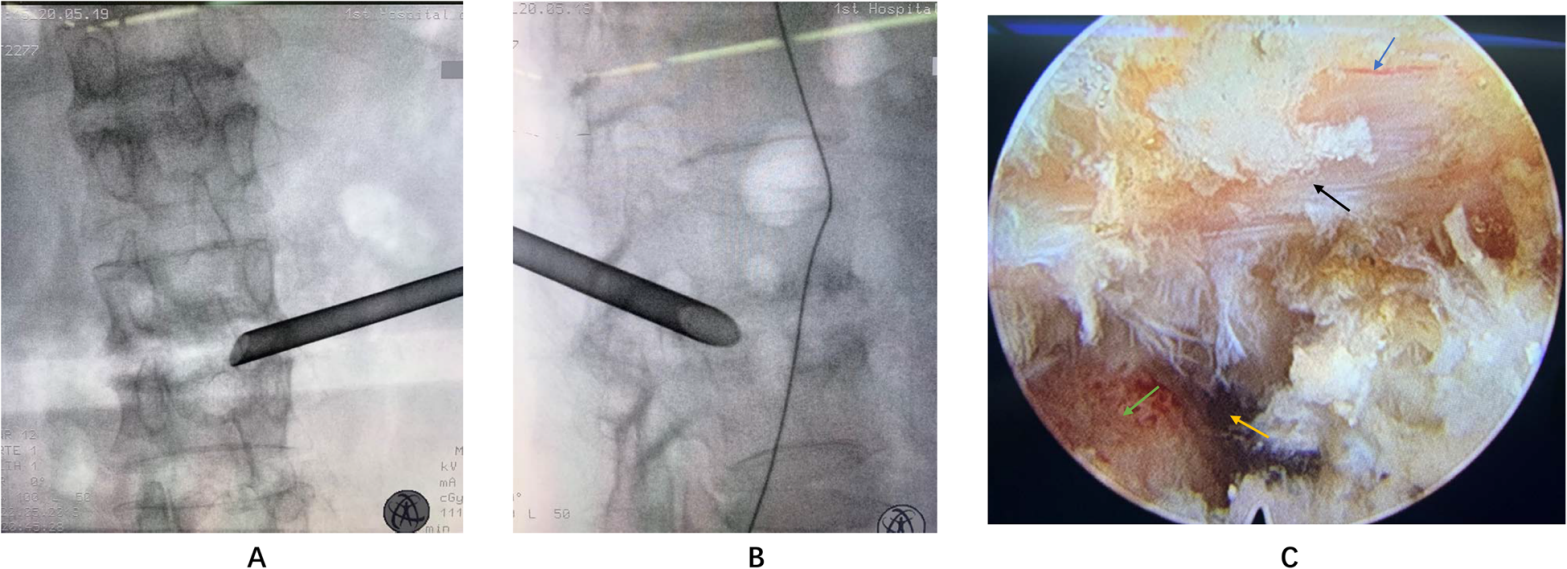
Surgical process **a** lumbar anteroposterior X-ray film. **b** lumbar lateral X-ray film. **c** view of PTED (blue arrow: nerve root; black arrow: ligamentum flavum; green arrow:end plate; yellow arrow: intervertebral space)

**Fig. 5:**
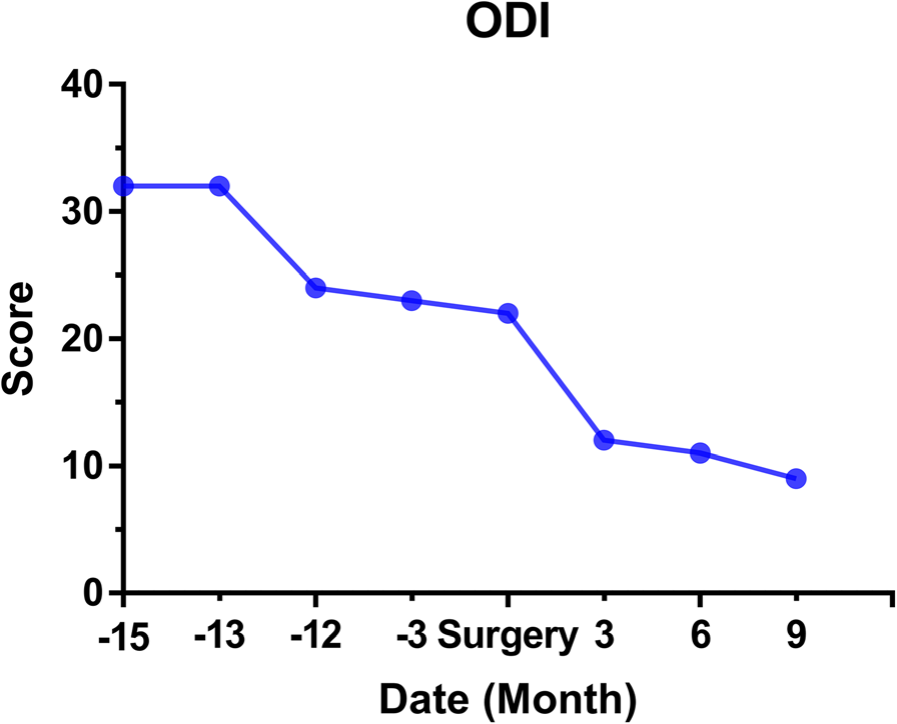
ODI evaluation

**Fig. 6:**
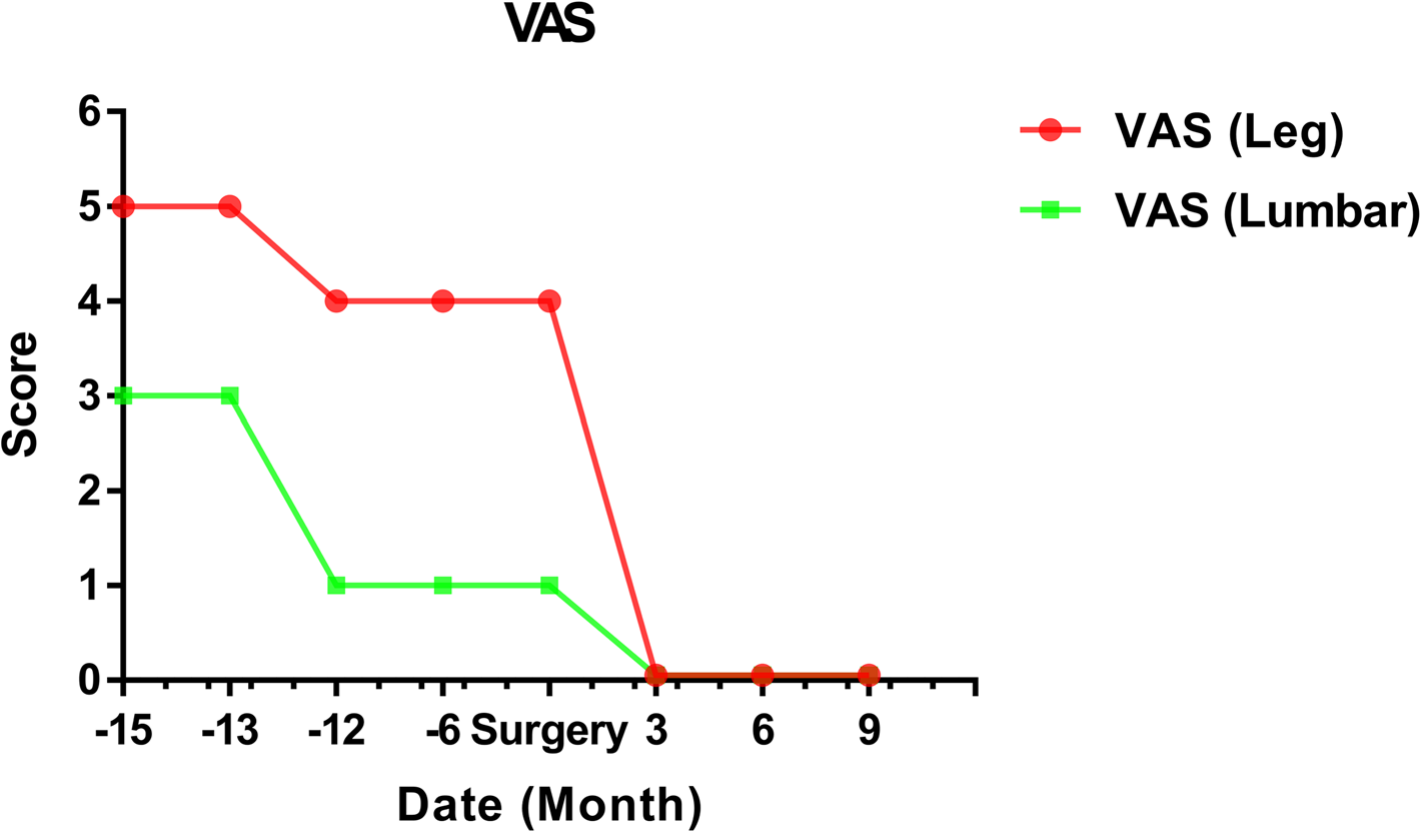
VAS evaluation

A 49-year-old man complained of lumbar back pain and weakness accompanied by radiating pain in inguinal and inner thigh region of right side for one and a half years. Lumbar Magnetic Resonance Imaging (MRI) taken in April 302,018 showed abnormal high signal intensity in the T12/L1 and L2/3 lumbar intervertebral discs with lateral recess stenosis (Fig. 1); His C-reactive protein (CRP) was higher and white blood cell (WBC) count was lower than normal all the time (Fig. 2). Blood fungus culture confirmed Aspergillusflavus infections. The patient was given itraconazole 15 ml/bid for 13 months before surgery. The lumbar back pain symptoms were relieved in a short term and no further damage was found in the vertebral body by MRI taken in Dec 11st, 2018 (Fig. 3). However, inguinal and inner thigh region radicular pain were not significantly improved in the one-year follow-up, so the patient came to our hospital in May 2019. One week later,we clean up the diseased tissue and made lateral recess plasty to expand the area of intervertebral foramen and relieve the radicular pain with PTED (Fig. 4). During the operation, some diseased tissues were taken for fungal culture. Meanwhile, antifungal agents (itraconazole) were injected into the intervertebral space. The pain in back and inguinal and inner thigh region of right side were ameliorated significantly after the operation. The patient was followed up for 9 months. His symptoms were improved significantly as shown by his ODI and VAS scores in Figs. 5 and 6.

## Discussion

The endplates have more blood supply than the inner annulus fibrosus and nucleus pulposus. Therefore, there might be two routes for lumbar intervertebral disc infection: 1. blood transmission; 2. direct contamination during operation [9]. Aspergillusflavus fungi were verified in this patient by blood culture. Most patients with fungal infections in intervertebral space have immunodeficiency diseases or long-term use of immunosuppressive drugs [10, 11]. However, none of the above-mentioned causes have been found in this patient. It is worth noticing that the patient has a history of liver cancer and his White Blood Cell (WBC) count was lower than normal. FeiMa and John W. Wilson mentioned that solid cancer could induce intervertebral fungal infection [8, 12]. It is speculated that the long history of liver cancer suppressed the patient’s immune function, thus leading to fungal infection of the spine.

Patients with fungal infection of the spine commonly have no obvious discomfort in the back at the early stage. The erosion of adjacent vertebral bodies or endplates gradually results in a decline in spinal stability. And some patients even suffer from radiating pain in the lower limb [13]**.** When the clinical symptoms occur, the condition has been developing for a long time, which then leads to a delayed diagnosis [14].

Because the patient had no significant improvement after taking itraconazole for 13 months as the conservative therapy, he expected surgical treatment. Considering the long-term fungal infection in his intervertebral space and the instability of his lumbar, our original plan was to fuse the diseased intervertebral space with cage and titanium bar for fixation. However, because of liver cancer, the patient’s condition was poor, and he could not afford the cost of fusion and fixation. So the surgical method was abandoned. After further discussion, we believed that lateral recess stenosis caused the radiating pain in the inguinal and inner thigh region of right leg. Finally, we decided that the preferred treatment was PTED for lateral recess plasty to expand the intervertebral foramen and relieve the pain.

Due to the history of liver cancer and poor general condition, this patient can not tolerate multiple operations. After careful preoperative study of MRI images, we found that both T12-L1 and L2-L3 had intervertebral height loss and intervertebral foramen stenosis with lateral recess stenosis and thought the pain symptoms were caused for both segments. During the operation, we first performed PTED surgery on L2-L3 and radiating pain in inguinal and inner thigh region of right side still existed after root block of L2-L3. Combined with MRI, we continued to perform T12-L1 segment. According to Hu Chaohui and He Zhong et al., using local antibiotics was effective in treating intervertebral space infection [15, 16]. Therefore, local antifungal drugs were injected into the lesion site. Postoperative follow-up for 9 months revealed that the patient’s inguinal and inner thigh region radiolurgic pain symptoms disappeared during activity.

Due to the long course of disease, the stability of intervertebral space was poor [17]. The patient was treated with gypsum vest fixation of the spine. After 9 months follow-up, the lumbar and inguinal and inner thigh region of right side pain was relived. This study confirmed that PTED is a good choice for the patient on antifungal agents for long-term fungal infection of the spine.

## Data Availability

The datasets used and/or analyzed during the current study are available from the corresponding author on reasonable request.

## Abbreviations

CRP: C-Creative protein
MRI: Magnetic Resonance Imaging
ODI: Oswestry Disability Index
PTED: Percutaneous transforaminal endoscopic discectomy
VAS: Visual Analogue Scale
WBC: White blood cells
